# Ratchet effect in veterinary antibiotic use by contract farmers from the perspective of production risk: Implications for public health

**DOI:** 10.3389/fpubh.2022.1008611

**Published:** 2022-09-16

**Authors:** Lingzhi Li, Ruiyao Ying

**Affiliations:** College of Economics and Management, Nanjing Agricultural University, Nanjing, China

**Keywords:** production risk, ratchet effect, agrochemical use reduction, public health, resistance to antibiotics

## Abstract

The current indiscriminate use of antibiotics for veterinary is irresponsible and misguided; it causes antibiotic resistance and adversely affects public health. The terms “habit” and “path dependence” are often used to explain the “excessive” use of agrochemicals. Yet, no research explored where the habit comes from and how it changes. This study investigates how veterinary antibiotic use changed with the production risk based on the multi-period production data set of 1,526 broiler contract farmers. The results show that the production risk has a ratchet effect on farmers' antibiotic use, leading to path dependence of farmers. Specifically, it showed a farmers' habit of steadily increasing antibiotic use and confirmed that the historical broilers' peak mortality was a key determinant to the continuation of this habit. It implies that higher the historical peak mortality, higher the current antibiotic use by farmers. Likewise, the impact of historical peak mortality on antibiotic use gradually increased with the farming experience. The increased historical peak mortality increased farmers' antibiotic use every time. Furthermore, large-scale farmers were more sensitive to historical peak mortality and therefore they increased antibiotic use excessively. The study suggests that improving farmers' production risk management capabilities, especially large-scale farmers, might help prevent extreme events. Moreover, this work contributes to the theoretical and empirical evidence on the ratchet effect, habit formation and farmers' antibiotic use and offers coherent insights for stakeholders to limit antibiotic use.

## Introduction

The excessive use of agrochemicals causes resistance, adversely affecting human and animal welfare, and it has been a significant problem in realizing environmental sustainability within Chinese agriculture. Bacteria and pests are resistant to antibiotics and pesticides, respectively (1), and these are transferable to the human body through living animals, animal products, and contaminated water and soil (2). The bacteriostatic effect of antibiotics for human use becomes weak with the excessive application, and thus health risks are increased. Estimates show that globally, over 700,000 people die of antibiotic resistance yearly (3). Likewise, the similar research project that by 2050 over 10 million people would die yearly from drug-resistant infections, if no serious action is taken to reduce antibiotic resistance.

Agrochemicals are intensively used in China. Ministry of Agriculture and Rural Affairs of the People's Republic of China (MARA-PRC) data reveal that the use of veterinary antibiotics per ton of animal products was 140 and 160 g in 2018 and 2019, respectively, which was much higher than the European Union (4). Trends show that from 1990 to 2018, the total pesticide application in China increased from 730,000 to 1.5 million tons, and pesticides increased from 4.9 to 9.1 kg/ha (5). Hence, a gradual agrochemical increase might threaten human health and sustainable development in the country (1). The Chinese government proposed two action plans to address these issues: (1) zero growth in pesticide use, and (2) reduction of veterinary antibiotic use. While these initiatives have a significant role in reducing antibiotic use, major knowledge gaps remain on whether and how they manifest green development in the agriculture sector.

Farmers' agrochemical use is generally linked to their habitual behavior of “being easy to increase but difficult to decrease.” Although extensive agrochemicals have many negative effects, farmers continue to increase their use (6). According to Cowan and Gunby (7), farmers have a low marginal propensity to adopt green alternatives due to the lock-in effect. Moreover, the risk aversion attitude and production risk perception make it difficult for farmers to reduce the use of agrochemicals (8). In a survey among 1,526 broiler farmers in Jiangsu Province, China, we found that the increase in veterinary antibiotic use was consistent with the historical peak mortality of broilers. Through qualitative interviews, we learned that after experiencing extreme mortality events, some farmers would continue heavy use of antibiotics due to fear of loss. Based on this, antibiotic use by farmers shows an obvious ratchet pattern—it seldom decreases and has a substantial chance of increasing. The term “ratchet effect” was proposed by Duesenberry (9). It refers to the impact of previous peak income and consumption experience on the actual consumption of individuals. Once formed, consumption habits are difficult to change, and consumption is easy to increase but difficult to decrease. Later, many researchers supported the habit-forming effects of resident consumption and pointed out that this effect determined the saving tendency of residents. For instance, Carroll et al. (10) demonstrated that the greater the influence of habit formation, the stronger the consumers' awareness of saving. Likewise, Harbaugh (11) pointed out that the memory of the great famine was the main reason for Chinese residents' tendency toward high savings, and that the intensity of the famine was positively correlated with their propensity to save. So far, a substantial literature complies the ratchet effect in labor market contexts and social dilemmas [e.g., (12)]. However, no evidence was found on whether a ratchet effect exists in farmers' antibiotic use, even though antibiotic use seems to have a ratchet pattern.

*Inter alia*, agricultural production risk is one of the main risks farmers face (13). It usually refers to the uncertainty in the yield and quality of agricultural products due to huge fluctuations in the factors such as temperature, rainfall, diseases, pests, and epidemics (14). Reducing production risk is the primary goal of farmers using agrochemicals. Evidence showed that the larger the mean or variance of historical output, the lower the farmers' enthusiasm to invest in agricultural production and the less willing they are to adopt new agricultural production technologies (15). However, regarding the habit-forming effects of antibiotic use, what should be considered is the impact of the historical peak mortality, rather than the relatively flat mean or variance of historical mortality. Thus, the important questions arise regarding the excessive veterinary antibiotic application and farmers' behavior. First, does there exist a ratchet effect in antibiotic use among Chinese farmers due to production risk? Second, if so, how large is the ratchet effect? Third, does it decrease with the increase in the farming experience? Last, is the rachet effect magnitude have a difference among different groups?

This study takes contract farmers in the broiler breeding industry – company + farmer – model to investigate the habit-forming effects of antibiotic use. The study significantly contributes to the prior literature in many ways. First, this pioneering work cues farmers' habit of using agrochemicals and investigates the causes. Second, it extends the application of the ratchet effect theory to farmers' antibiotic use and provides a new analytical perspective and robust explanation of over-reliance on agrochemicals. Further, this study helps clarify farmers' decision-making mechanism of agrochemicals and coherent policy actions to promote green alternatives and reduce antibiotic resistance risks to human and animal health.

## Literature review and hypothesis

Many studies explored the factors of the over-reliance on agrochemicals among Chinese farmers. Representative academic views include risk aversion, insufficient information, and habits. It is widely agreed in the academic community that farmers use agrochemicals in large quantities to avoid production risks. The unanimous conclusion from previous studies indicates that the more risk-averse the farmers, the higher the use of agrochemicals (16). Similarly, farmers' access to information is an important factor affecting agrochemicals' use and vice-versa (17). Most farmers in developing countries cannot obtain timely and accurate technical information and can only decide on pesticide use from their experience (18). Dasgupta et al. (19) regarded the excessive use of agricultural chemicals as a bad habit and examined whether farmers had bad antibiotic habits based on income and farm ownership. Studies also discussed the habits of pesticide types selection, application frequency, and compliance with the instructions, concluding that farmers tended to use the same types of pesticides for years (20), apply pesticides multiple times in a short period (21), and use pesticides above the recommended doses specified in the instructions (22). These bad habits can cause farmers to overuse pesticides (23). In general, previous studies directly named farmers' behaviors as habits, but did not thoroughly investigate the origin and size of habits.

Habit formation asserts that the utility of current behavior is related to past behavior. In the literature, the term “behavior” usually refers to consumption. Unlike the traditional utility function, the function under habit formation is inseparable in time; hence, the utility of current consumption is related to the weighted average of consumption in previous periods. Thus, ratchet effect explains the origin of consumption habits; previous peak income shape consumption habits, and leads to consistency and continuity between current and previous consumption (9). Therefore, when current income decreases, consumption does not decrease immediately. Individuals would rather reduce savings or borrow money to maintain the original consumption level. However, previous studies usually use consumption in the previous period to represent consumption in various past periods and focus on evaluating the impact of consumption in the previous period on current consumption [e.g., (24)], lacking discussion on the impact of previous peak income. If consumption in the previous period impacts current consumption, the residents have consumption habits. However, where do the consumption habits come from? Consumption in the previous period did not come out of thin air. Therefore, verifying the habit-forming effects of previous peak income is reasonable. A few exceptions, only Corrales and Mejías's (25) work on Latin America incorporated the ratio of current income to previous peak income into the model to examine the ratchet effect of marginal propensity to consume based.

Based on the prior debate on the connection of the ratchet effect with consumption, this study investigates the ratchet effect's influence on farmers' antibiotic use. The farmers' consumption and/or application of agrochemicals has inertia, which may originate from the historical peak mortality. The discussion of the impact of historical peak mortality is similar to that of extreme events. Extreme events often have a long-lasting impact. For example, the experience of hunger in childhood causes great fear in children. Even if they no longer face the real danger of hunger in adulthood, they still cherish food and money exceptionally and tend to increase savings (11). The high loss experience may prompt farmers to form a habit of heavy antibiotic use. The logic is that the memory of loss may cause irrational preventive antibiotic use, and farmers choose to give up part of their profits to avoid losses as much as possible. Individuals tend to imitate past successful behavior patterns, even if the environment has changed (26). Increasing antibiotic use in this state of mind increases their sense of security. Hence, such a loss experience, to a certain extent, further causes sudden and virulent infectious diseases. Given these, the following hypothesis is proposed: The extreme mortality events (i.e., high historical peak mortality), instigate farmers' current excessive antibiotic use. In other words, after an extreme mortality event occurs, farmers' antibiotic use remains relatively high; thereby, the current antibiotic use is consistent with that in the previous period.

## Antibiotic use by contract farmers

### Data source

The longer-term input and output panel data is considered ideal for studying farmers' behavior of antibiotic use habits. In this study, contract farmers in broiler industry “company + farmer” model were used to acquire sufficient data while maintaining the sample's representativeness.

First, regulations for the broiler industry in China have been raised due to environmental protection policies and technological requirements. As a result, more small and medium-sized farmers have left this industry, and the farming scale and industrialization have continued to increase (27). From 2004 to 2017, the number of farms with an annual output of 2,000–10,000 broilers was reduced by half, while the number of farms with an annual output of more than 50,000 broilers increased by four times (28). Meanwhile, leading companies have increased and are gaining an increasing market share. In 2019, China's total broiler output was 9.3 billion, a quarter of which (2.258 billion) was produced by five listed broiler companies. The sample company in this study is one of the five listed companies. It has 22 fully-owned subsidiaries, mainly located in Jiangsu and Anhui provinces. Contract farmers with this company should represent the fundamentals of broiler farmers in China.

Second, research on production risk requires long-term historical data. In the commissioned farming model, the sample subsidiaries have detailed input and output data at the farmer–chicken house level, enabling the data robustness of this study. According to the contract, the company provides farmers with chicks, feed, medicine, training, and technical guidance, checks the quality, and accepts grown broilers. Farmers build sheds, pay deposits, and carry out broiler farming according to the company standards. The company sets the prices of materials and grown broilers. Farmers receive materials from the company on a credit basis and receive payment of gross profit from the company after the delivery of grown broilers. Farmers have a certain degree of discretion in using antibiotics; they can obtain and use antibiotics by company's recommended dosage as long as they meet the withdrawal time and residue requirements. Although the company mainly bears the market risk, farmers also bear part of the farming risk. They need to improve farming performance through excessive antibiotics and appropriate management. Therefore, there is a great difference in antibiotic use among farmers. In other words, there is no lack of heterogeneity between contract farmers. The data covers several variables: number of chicks, mortality, cost of antibiotics, cost of vaccines, and cost of disinfectants. The companies provided the individual characteristics of farmers, such as age and years of farming experience.

The sampling period was from January 2016 to June 2018. During this period, 1,526 farmers had farming contracts with the sample subsidiaries and delivered more than 2 batches of broilers. Under normal circumstances, farmers produce 3 batches of broilers per year. Over one-third of the sampled farmers produced 7–10 batches of broilers. A small proportion, less than 10% farmers, produced more than 11 batches of broilers. The costs of antibiotics and other production factors were deflated by the producer price index of agricultural products of live poultry in 2016. The price index data comes from the China Statistical Yearbook 2017–2019.

### Antibiotic use by farmers

**Figure 1:**
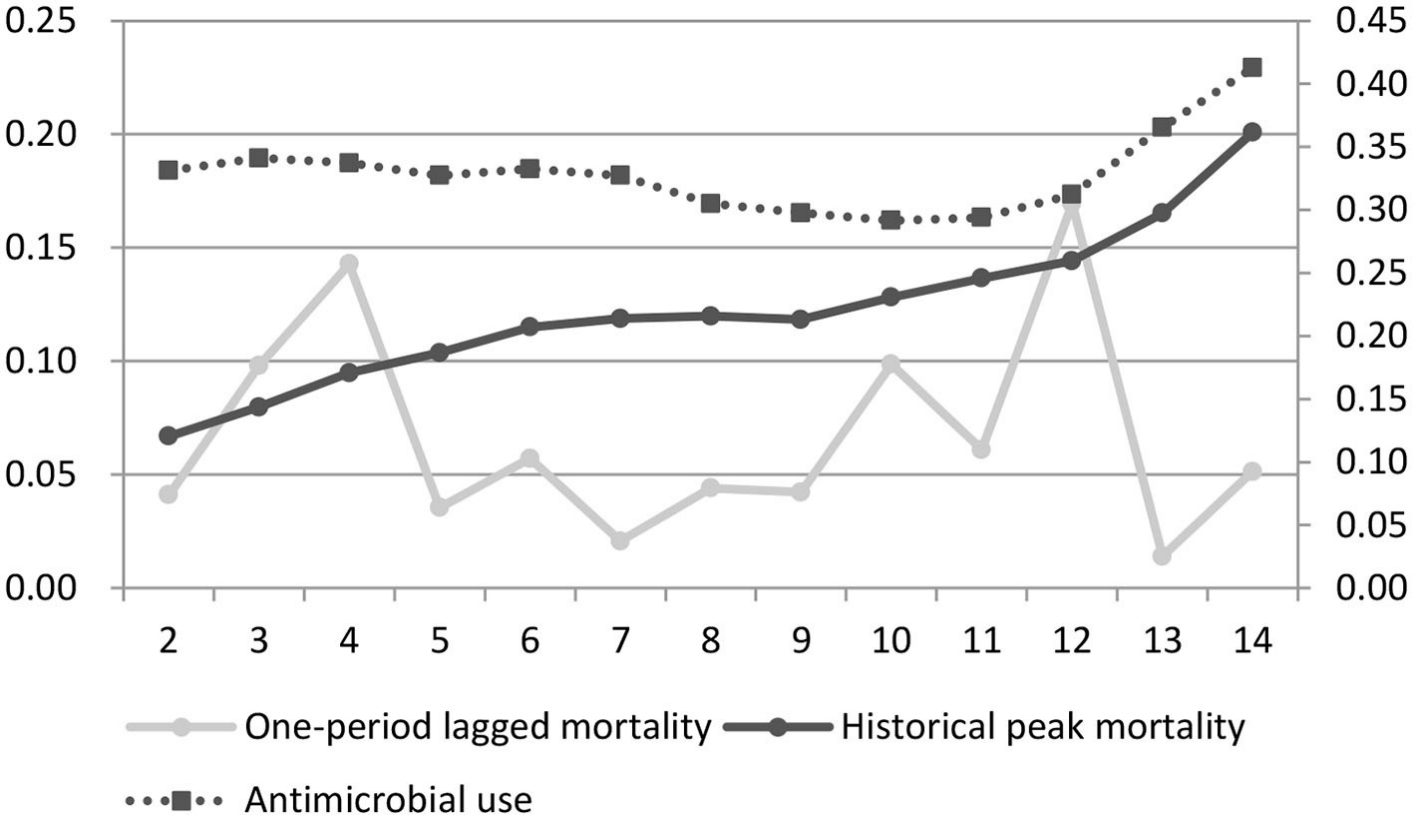
Broiler mortality and current antibiotic use among the sample farmers. The horizontal axis represents batches of broilers, the left vertical axis represents the mortality, and the right vertical axis represents the cost of antibiotics per broiler (yuan/broiler).

Figure 1 depicts the overall changes in antibiotic use, one-period lagged mortality, and historical peak mortality by batch during the sample period among the sample farmers. In general, the historical peak mortality showed a increasing trend, and antibiotic use changed consistently with the historical peak mortality. In contrast, one-period lagged mortality was volatile and did not significantly correlate with antibiotic use. In detail, when less than 10 batches were bred, the increase in historical peak mortality was small (probably since extreme events were unprecedented), and antibiotic use fluctuated but remained stable overall. As time passed, the historical peak mortality increased sharply, and so did antibiotic use. Even though one-period lagged mortality sharply decreased, antibiotic use remained high. We can also infer from the changing trend that historical peak mortality had a threshold effect on antibiotic use, as antibiotic use increased only when the historical peak mortality was high enough.

**Figure 2:**
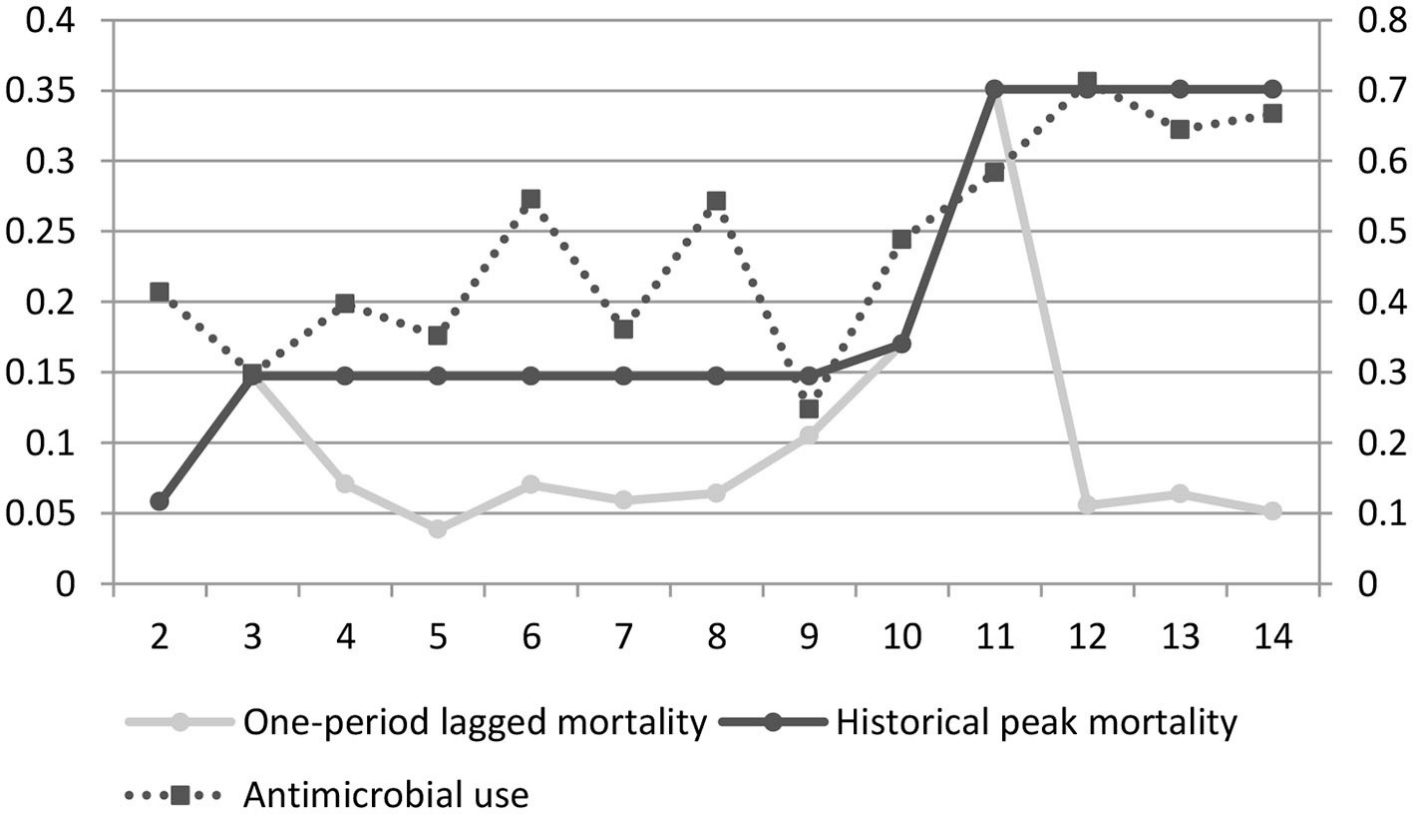
Broiler mortality and current antibiotic use by an individual farmer. The horizontal axis represents batches of broilers, the left vertical axis represents the mortality, and the right vertical axis represents the cost of antibiotics per broiler (yuan/broiler).

The relationship between mortality and antibiotic use is clearer in individual farmers. As shown in Figure 2, the farmer experienced three increases in historical peak mortality during the sample period. The first increase lasted a long time, from period 3 to 9. During this period, antibiotic use fluctuated slightly, mainly due to many disturbance factors and consequent high uncertainty in agricultural production. The second increase was small, lasted only one period, and is not discussed here. However, the third increase was very large and lasted until the end of the sample period. During this period, antibiotic use and historical peak mortality increased sharply and remained high even when the one-period lagged mortality decreased significantly. After experiencing extreme mortality events, farmers may desire more to avoid losses and maintain low mortality, thus making continued heavy use of antibiotics. It suggests that this phenomenon is common. Therefore, it can be predicted that the production risk has a ratchet effect on antibiotic use by farmers.

Extreme mortality events have randomness (for example, due to an exogenous sudden temperature drop that catches farmers unprepared) and regularity. Generally, farmers with more years of farming experience are more experienced, and those with a larger farming scale have more capital and technology (29). So, is the regularity of extreme mortality events reflected in the lower historical peak mortality for farmers with more years of farming experience or a larger farming scale?

**Figure 3:**
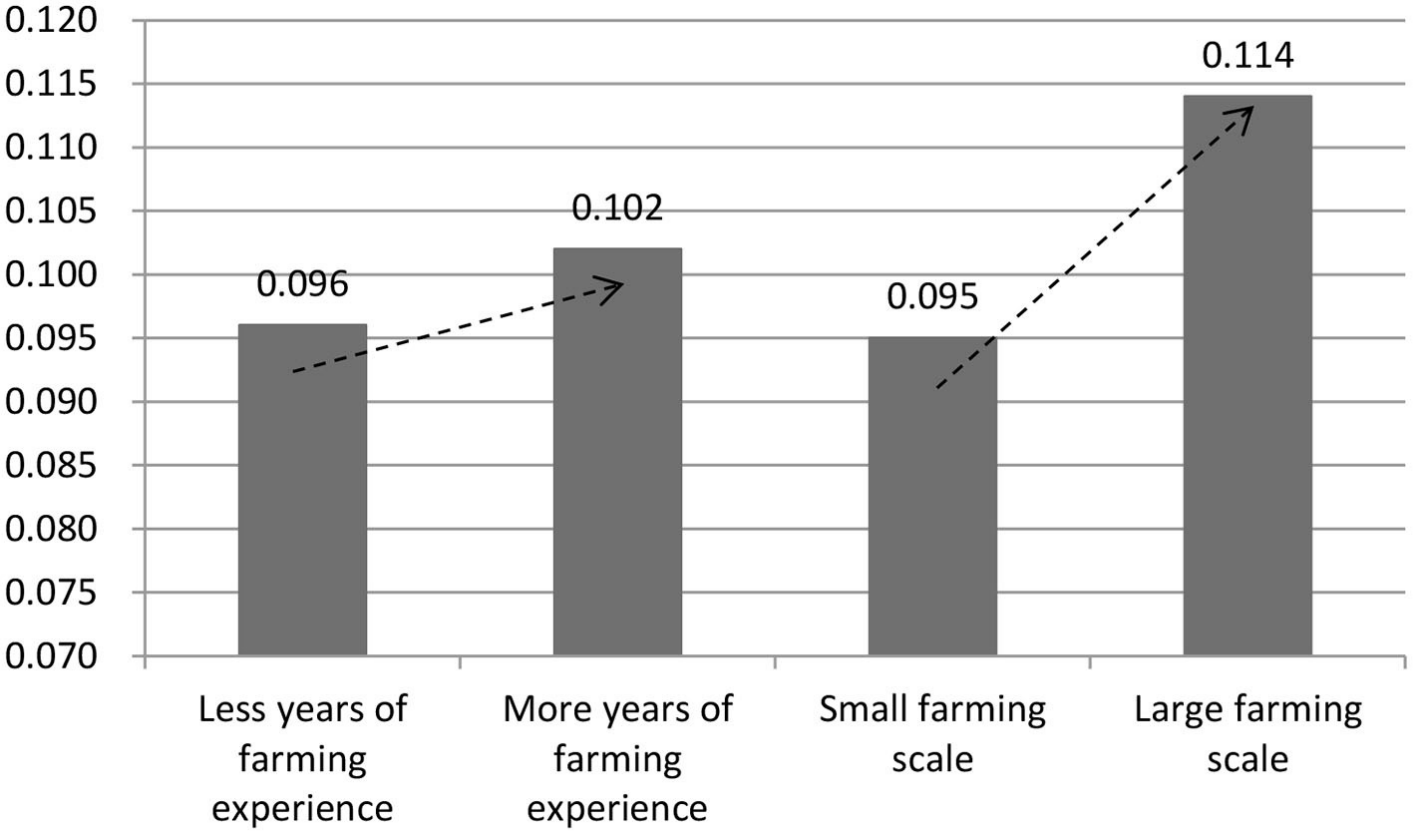
Broiler mortality and current antibiotic use by an individual farmer.

The data suggests, as shown in Figure 3, that this may not be the case. There was no significant difference in peak mortality between farmers with fewer years of farming experience and those with more years of farming experience. Thus, the severity of extreme mortality events was weakly correlated with years of farming experience. This reflects the exogeneity and randomness of extreme mortality events. Extreme mortality events can occur to both beginner and experienced farmers. In contrast, historical peak mortality was associated with the farming scale. Compared with small-scale farmers, large-scale farmers experienced higher peak mortality; they experienced more severe extreme mortality events. Hence, the ratchet effect of antibiotic use may be more pronounced among large-scale farmers. Section Heterogeneity examines the ratchet effect in different groups of farmers.

## Sample, model, and variables

### Model specification

In analyzing the ratchet effect in consumption, the current marginal propensity to consume depends on the relative values of current income and previous peak income, and the lagged marginal propensity to consume (30). Therefore, the following model is proposed to investigate the ratchet effect in antibiotic use by farmers:


(1) $$\begin{array}{r} Y_{it}\;=\;\alpha_{0}+\alpha_{1}Risk_{it,peak}+\alpha_{2}Risk_{it,m}+\alpha_{3}Y_{i,t-1}+\alpha_{4}X_{it}+\varepsilon_{it} \end{array}$$

where *Y*_*it*_ is the antibiotic use in period *t*; *Risk*_*it, peak*_ is the peak mortality before period *t*, with the coefficient α_1_ representing the strength of the ratchet effect; *Risk*_*it, m*_ is the mean mortality before period *t*; *Y*_*i, t*−1_ is the one-period lagged antibiotic use, with the coefficient α_2_ representing the strength of the habit-forming effects; *X*_*it*_ is other factors that affect antibiotic use in period *t*, such as the age of household head, years of farming experience, farming scale, current vaccine input, disinfectant input, and rearing density; and ε_*it*_ is the random error term. Fixed effects of broiler breed, chick-receiving month, and chick-receiving year were also included in the model to control unobservable factors.

Model (1) is essentially a dynamic panel data model as the antibiotic use is affected by the antibiotic use in the previous period. Naik and Moore (31) suggested using fixed effects estimation for this model. They believed that fixed effects could eliminate individual heterogeneity and the impact of previous behavioral characteristics not captured by one-period lagged variables. However, fixed-effects estimation is questioned in two ways: First, although fixed effects control for unobservable heterogeneity that does not change with time between individuals, they cannot address endogeneity caused by omitted variables that change with time. Second, fixed-effects dynamic panel models are biased in finite samples (32).

To this end, the System Generalized Method of Moment (GMM) approach was used to test robustness in this study. System GMM corrects the bias of fixed effect estimates in finite samples and is robust to weak instruments. It uses moment conditions in both difference and level equations and the first differences of lagged variables as instruments for level variables in the level equation, thus being more effective than difference GMM estimation.

### Variable definitions and descriptive statistics

Since production risks are almost always related to adverse events that have not yet occurred and are hypothetical, therefore cannot be directly observed (33). A few researchers measured this risk by risk perception (34), but this approach has drawbacks, such as subjectivity and strong endogeneity. Some researchers used objective output data to represent current and/or future production risks. An early representative study by Anderson and Griffiths (35) examined the effect of production risk on inputs by measuring the mean and variance of crop yields. In addition to the mean and variance of output, Falco et al. (36) also measured downside risk to output, expressed by the skewness of output. A decrease in output skewness implies an increased downside risk to output, which is an increased probability that the output is below the mean given the mean and variance (36, 37). Building on the existing literature, this study measures production risk by historical peak and mean mortality, assesses the ratchet effect, and examines the impact of historical peak mortality on farmers' current antibiotic use. In period *t*, the historical peak mortality is the highest mortality experienced by farmers before period *t*.

The definitions and descriptive statistics of key variables in the model are shown in Table 1. The average age of the sampled farmers was 45 years. They generally had 5 years of farming experience and a mean farming capacity of nearly 20,000 broilers per batch. The broilers were divided into fast, medium and slow-growing, with an average rearing period of 61, 80, and 94 days, respectively. Most farmers raised medium-growing broilers, followed by slow-growing. In addition to the use of antibiotics, disease prevention measures implemented by farmers as required by the company included vaccination and disinfection.

**Table 1:** Variable definitions and descriptive statistics.

**Variable**	**Definition**	**Mean**	**Standard deviation**
Antibiotic use	Current antibiotic cost (yuan/broiler)	0.334	0.135
Historical peak mortality	The highest mortality in the past (number of deaths/number of chicks received)	0.097	0.079
Historical mean mortality	Mean mortality in the past	0.063	0.037
One-period lagged antibiotic use	One-period lagged cost of antibiotics (yuan/broiler)	0.330	0.129
Age	Age of household head (years)	44.961	7.214
Years of farming experience	Years of engaging in contract farming	5.153	2.782
Scale of farming	Current number of chicks received (10 thousands)	1.776	1.078
Vaccination	Current vaccination cost (yuan/broiler)	0.191	0.072
Disinfectant use	Current disinfectant cost (yuan/broiler)	0.018	0.017
Rearing density	Current number of chicks/housing area (broilers/square meter)	12.273	2.400
Medium-growing broilers	Whether the breed is medium-growing broilers (1 = yes; 0 = no)	0.460	0.498
Fast-growing broilers	Whether the breed is fast-growing broilers (1 = yes; 0 = no)	0.303	0.460

## Empirical analysis

### General results

As shown in Table 2 (column 1), the ratchet effect in antibiotic use was estimated by System GMM. Two-step estimation was used for regression. Two and three-period lagged antibiotic use were instrumental variables for one-period lagged antibiotic use. For standard deviations in the two-step estimates, finite-sample corrections were made per Windmeijer (38) to correct possible downward bias. Sargan's test of System GMM estimates suggests that the instruments are generally valid. The test for residual serial correlation indicates no second-order serial correlation in the differenced residuals. Thus, it can be concluded that there is no serial correlation in the error term of the original model. The *p*-value of the Wald test indicates that the model is overall significant.

**Table 2:** The impact of historical peak mortality on current antibiotic use.

	**Current antibiotic cost (yuan/broiler)**	
	**(1)**	**(2)**
Historical peak mortality	0.140**	0.023
	(0.070)^a^	(0.030)
Historical mean mortality	−0.239	0.247***
	(0.176)	(0.072)
One-period lagged antibiotic use	0.144***	−0.005
	(0.029)	(0.013)
Age	−0.013	0.036***
	(0.009)	(0.006)
Years of farming experience	−0.003	−0.048***
	(0.010)	(0.006)
Scale of farming	−0.003	−0.004
	(0.005)	(0.003)
Vaccination	0.041	−0.027
	(0.029)	(0.022)
Disinfectant use	−0.011	0.128
	(0.118)	(0.079)
Rearing density	0.002*	0.002***
	(0.001)	(0.001)
Fixed effect^b^	Yes	Yes
Constant term	0.834**	−1.099***
	(0.350)	(0.248)
Sample size	8,045	8,045
Wald test	157.71***	—
*R*-squared	—	0.034
Number of farmers	1,526	1,526
Estimator	System GMM	Fixed effect

^a^Numbers in parentheses are the standard errors of the estimated coefficients. *, **, *** indicate significance at the 10%, 5%, and 1% levels, respectively. ^b^Fixed effects include breed, month, and year fixed effects. Correlation coefficients are not listed due to space limitations. The same applies to the following tables.

As shown in column (1), the coefficient of historical peak mortality is significant and positive at the 5% level. Specifically, each additional unit of the historical peak mortality was associated with an increase of 0.140 yuan/broiler in current antibiotic. Chah et al. (39) stated that farmers were not ready to risk losing their chickens and the main concern about production risks contributed to the farmers' heavy use of antibiotics. In addition, the impact of one-period lagged antibiotic use on current antibiotic use was significant and positive at the 1% level (0.144). The above results indicate that antibiotic use by Chinese farmers has a strong path dependence on historical peak mortality and also shows a significant habit-forming effect, thus confirming a ratchet effect in antibiotics use.

Moreover, only the coefficients of historical peak mortality, one-period lagged antibiotic use, and rearing density are significant and positive. Intensive farming generally suffers from high stocking densities, increasing the risk of disease transmission (40). Therefore, reducing rearing density is an effective way to reduce antibiotic use. The historical mean mortality had no significant effect on current antibiotic use. It suggests that farmers are more sensitive to extreme mortality events than the average historical mortality level.

Table 2 also reports the regression results for the fixed effects. As shown in column (2), the coefficients of historical peak mortality and one-period lagged antibiotic use are no longer significant, and the latter is negative. With relatively few years of panel data, the fixed-effect estimates of the lagged explained variables are biased downwards (41). Therefore, the endogeneity of habit formation cannot be ignored.

Next, the continuous historical peak mortality was transformed into a series of dummy variables based on a certain “threshold” value. It helps determine how high the historical peak mortality needs to change the current antibiotic use, resulting in a ratchet effect. Determining this value guides apposite practice. Companies can predict the trend of antibiotic use by farmers according to the mortality, and develop accurate antibiotic use reduction plans in advance. The “threshold” values selected in this study are 0.10, 0.11, 0.12, 0.13, 0.15, 0.17, 0.19, and 0.21. The first value, 0.10, is slightly above the mean historical peak mortality, 0.11 is the 75th percentile of historical peak mortality, and 0.21 is close to the 95th percentile. The results are shown in Table 3. If the historical peak mortality value is higher than 0.13, it might have a significant positive impact on the current antibiotic use^1^. The value of 13% is the 94th percentile of one-period lagged mortality, which can be considered an extreme mortality event.

**Table 3:** Threshold effect of historical peak mortality on current antibiotic use.

	**Current antibiotic cost (yuan/broiler)**							
	**(1)**	**(2)**	**(3)**	**(4)**	**(5)**	**(6)**	**(7)**	**(8)**
(A certain value)^b^	0.10	0.11	0.12	0.13	0.15	0.17	0.19	0.21
Historical peak mortality above a certain value	0.001	0.008	0.009	0.022*	0.035**	0.052**	0.060**	0.060**
	(0.009)	(0.009)	(0.010)	(0.012)^a^	(0.013)	(0.015)	(0.017)	(0.019)
Historical mean mortality	0.015	−0.085	−0.085	−0.143	−0.184	−0.216	−0.235	−0.223
	(0.150)	(0.155)	(0.153)	(0.153)	(0.154)	(0.147)	(0.146)	(0.147)
One-period lagged antibiotic use	0.128**	0.138**	0.137**	0.140**	0.142**	0.144**	0.142**	0.140**
	(0.030)	(0.030)	(0.030)	(0.029)	(0.029)	(0.029)	(0.029)	(0.029)
Control variable	Yes	Yes	Yes	Yes	Yes	Yes	Yes	Yes
Fixed effect	Yes	Yes	Yes	Yes	Yes	Yes	Yes	Yes
Sample size	8,045	8,045	8,045	8,045	8,045	8,045	8,045	8,045

^a^Numbers in parentheses are the standard errors of the estimated coefficients. *, ** indicate significance at the 10%, 5%, and 1% levels, respectively. ^b^The mean historical peak mortality was 0.097, with a 75th percentile of 0.111, a 90th percentile of 0.168, and a 95th percentile of 0.219.

How much economic loss do farmers incur with mortality higher than 13%? According to the settlement data provided by the companies, the average production cost per broiler (including only direct materialized costs, such as costs of chicks, feed, and medicines) was 19.102 yuan, the average selling price per broiler was 21.469 yuan, and the average gross profit per broiler was 2.368 yuan. To simplify the analysis, it is assumed that broilers die just before being delivered as grown broilers. In this case, the gross profit is reduced to 0 yuan when the mortality reaches 11%. If labor, fixed asset depreciation, fuel, and water and electricity costs (approximately 1 yuan/broiler in total) are deducted, farmers would already suffer great losses. Assuming that broilers die in the middle of the production process, the average production cost per dead broiler would be approximately 9.551 yuan. In this case, the gross profit is reduced to 0 yuan when the mortality reaches 20%. Therefore, a higher than 13% mortality is likely to cause farmers' profits to drop below zero. This study indicates that the experience of fruitless labor in farming can lead farmers to over-rely on antibiotics to reduce production risks.

### Intertemporal changes in the ratchet effect

If the ratchet effect is long-standing, it causes high resistance to reducing antibiotic use. Therefore, it is necessary to explore the persistence of the ratchet effect. To this end, a historical peak mortality duration variable is created, and the interaction between the historical peak mortality and duration is added to the basic model. After a peak mortality occurred, the historical peak mortality would remain unchanged unless new higher mortality occurred. The data shows that three-fifths of the sample farmers experienced 1–2 stepwise increases in historical peak mortality during the sample period. On average, peak mortality was replaced by a higher mortality after 4–5 periods. If the historical peak mortality occurred in the previous period, the duration variable takes the value 1; if the historical peak mortality occurred in the period before last, the duration variable takes the value 2 and so forth.

In addition, the continuous duration variable was transformed into a series of dichotomous variables based on a certain “threshold” value to examine further the short-term and long-term effects of historical peak mortality. Specifically, the dichotomous variables are whether peak mortality lasts for 2 periods or more, likewise repeated for 2–6 periods.

The results are shown in Table 4. As shown in column (1), the interaction between historical peak mortality and duration is significant and positive. It indicates that the impact of historical peak mortality on current antibiotic use increases with the increase in farming experience. The duration is a dummy variable in columns (2)–(6). When the duration exceeds 5 periods, the interaction coefficient between historical peak mortality and duration is large and significant (positive) at the 1% level. It indicates that historical peak mortality lasting for more than 5 periods leads to increased antibiotic use. Therefore, the longer the duration of the historical peak mortality, the higher the antibiotic use. The possible reason is that the longer the duration, the higher the value of the historical peak mortality. In other words, the duration indirectly reflects the historical peak mortality level.

**Table 4:** Intertemporal changes in the impact of historical peak mortality on current antibiotic use.

	**Current antibiotic cost (yuan/broiler)**				
	**(1)**	**(2)**	**(3)**	**(4)**	**(5)**	**(6)**
		*T* = 2	*T* = 3	*T* = 4	*T* = 5	*T* = 6
Historical peak mortality	0.055	0.037	0.107	0.140**	0.138**	0.131*
	(0.071)	(0.073)	(0.073)	(0.072)	(0.064)	(0.068)
Historical peak mortality * duration^b^	0.060***					
	(0.016)^a^					
Historical peak mortality * lasting for more than T periods		0.136***	0.070*	0.050	0.204***	0.190***
		(0.035)	(0.042)	(0.052)	(0.056)	(0.066)
Historical mean mortality	0.121	0.046	−0.130	−0.219	−0.201	−0.208
	(0.166)	(0.178)	(0.182)	(0.173)	(0.169)	(0.170)
One-period lagged antibiotic use	0.164***	0.151***	0.144***	0.150***	0.159***	0.150***
	(0.028)	(0.029)	(0.030)	(0.030)	(0.028)	(0.029)
Control variable	Yes	Yes	Yes	Yes	Yes	Yes
Fixed effect	Yes	Yes	Yes	Yes	Yes	Yes
Sample size	8,045	8,045	8,045	8,045	8,045	8,045

^a^Numbers in parentheses are standard errors. *, **, *** indicate significance at the 10%, 5%, and 1% levels, respectively. ^b^“Duration” indicates how long the historical peak mortality in period t lasts. “Lasting for more than T periods” is a dummy variable. If the historical peak mortality in period t lasts for T periods or longer, it takes the value 1, otherwise, it takes 0.

Notably, the one-period lagged mortality was lower than the historical peak mortality during the duration. Even if the one-period lagged mortality is low, antibiotic use does not decrease and might increase further. This more rigorously verifies the ratchet effect proposed by Duesenberry (9) with the characteristics of “being easy to increase but difficult to decrease.”

Results in Table 2 conclude that the higher the historical peak mortality, the higher the current antibiotic use. Combining these with Table 4, farmers increase antibiotic use to a higher level every time the historical peak mortality increases. Hence, these findings rigorously explore habit formation and provide strong empirical evidence for understanding farmers' antibiotic use.

### Heterogeneity

Important characteristics of farmers include age, education, years of farming experience, the scale of farming, and the number of household laborers (17, 43). Due to data limitations, the characteristics discussed in this study are age, years of farming experience, and scale of farming. Older farmers are more risk-averse and may be more sensitive to historical peak mortality (44). Due to the learning effect, farmers with longer broiler experience are less sensitive to historical peak mortality. In theory, large-scale farmers have relatively high-risk tolerance and might be more insensitive to historical peak mortality.

We tested that heterogeneity by adding the interactions of age, years of farming experience, and farming scale with historical peak mortality to the basic model, are illustrated in Table 5. The impact of historical peak mortality on current antibiotic use is not related to age and farming experience, but only depends on the scale of farming. Specifically, for each additional unit of historical peak mortality, antibiotic use by large-scale farmers increased by 0.299 yuan/broiler, while that by small-scale farmers only increased by 0.177 yuan/broiler. There was no heterogeneity in the effect of historical peak mortality by age and/or years of farming experience. It might be conferred that farmers of different ages or years of farming experience did not experience significant differences in historical peak mortality.

**Table 5:** Different effects of historical peak mortality on different categories of farmers.

	**Current antibiotic cost (yuan/broiler)**		
	**(1)**	**(2)**	**(3)**
Historical peak mortality	0.138*	0.160**	0.129*
	(0.073)^a^	(0.074)	(0.068)
Historical peak mortality * whether being older than 50 years^b^	0.016		
	(0.116)		
Historical peak mortality * whether having more than 7 years of farming experience		−0.075	
		(0.082)	
Historical peak mortality * whether having more than 25,000 chicks			0.129**
			(0.060)
Historical mean mortality	−0.239	−0.251	−0.249
	(0.175)	(0.175)	(0.175)
One-period lagged antibiotic use	0.144***	0.144***	0.142***
	(0.029)	(0.029)	(0.029)
Control variable	Yes	Yes	Yes
Fixed effect	Yes	Yes	Yes
Sample size	8,045	8,045	8,045

^a^Numbers in parentheses are the standard errors of the estimated coefficients. *, **, *** indicate significance at the 10%, 5%, and 1% levels, respectively. ^b^“Whether being older than 50 years” takes the value 1 if the age of household head is 50 years and above, otherwise it takes 0. “Whether having more than 7 years of farming experience” takes the value 1 if the household head had more than 7 years of farming experience, otherwise it takes 0. “Whether having more than 25,000 chicks” takes the value 1 if the number of chicks received by the farmer was 25,000 or more, otherwise it takes 0. c. 50 years of age, 7 years of experience, and 25,000 chicks are the 75th percentiles of the corresponding variable. Farmers with more than 25,000 chicks usually own more than two chicken houses.

The key results show that farming scale negatively affect antibiotic use, although not significant. This is consistent with numerous studies discussing the relationship between farming scale and pesticide use (23, 45). The reasons for farming scale to promote the reduction of agrochemical use are multi-dimensional. On the one hand, with the increase in farming scale, farmers choose to reduce agrochemical use to reduce material costs. On the other hand, large-scale farmers are more likely to carry out scientific production and management, can better control agrochemical use, and implement more efficient and effective disease prevention and control (45). Furthermore, large-scale farmers are more able to adopt advanced production technology (29), and reduce the incidence of disease by optimizing the production environment, thereby reducing the use of agrochemicals.

However, this study further shows that large-scale farmers use fewer agrochemicals and are more sensitive to historical peak mortality. Although large-scale farmers have a higher risk appetite than small-scale farmers, they are still risk-averse. Large-scale farmers have experienced greater production losses with the same historical peak mortality, which may lead to greater sensitivity to historical peak mortality.

## Conclusions

Antibiotic resistance and poultry disease transmission incidents to humans have increased several-fold. Farmer excessively uses antibiotics to reduce mortality rates and increase feed efficiency. A better understanding of the farmers' decision makings of antibiotic use in the production process is necessary for reducing antibiotic use and ensuring the footprints of animal production on human health. This study empirically proves the ratchet effect on farmers' antibiotic application using a data set of 1,526 contract farmers from 2016 to 2018. The study findings offer in-depth understanding of habit formation, ratchet effect, and veterinary antibiotics use and provide cues for policy and practice.

The findings offer insights into theoretical and empirical literature on habit formation and the ratchet effect in many ways. First, the results showed that the historical peak mortality significantly (positively) affected current antibiotic use, which did not decrease with the farming experience. In other words, significant ratchet effects can occur irrespective of age and experience level as long as they operate on a large scale. Likewise, findings further confirm that Large-scale production is a general trend in the farming industry. Second, the one-period lagged antibiotic use also significantly and positively affected current antibiotic use. Therefore, due to production risks, farmers have a ratchet effect in antibiotic use. As the historical peak mortality increased in a stepwise manner, farmers' antibiotics followed a similar pattern. Third, the historical peak mortality had a greater positive effect on antibiotic use by larger-scale farmers. Larger-scale farmers that experienced high mortality events would maintain higher antibiotic use for a long time. Further, the study extends the application of “Ratchet effect theory” by studying the antibiotic use behavior of broiler farmers.

Based on the findings, we proposed coherent policy actions for farmers, contract companies, the government, and other stakeholders linked to the broiler breeding industry. First, there is a need to effectively improve farmers' production risk management capabilities to reduce the antibiotic resistance risks to animal and human health. For the farmers, efforts should be made to encourage them to enhance farming conditions and sanitation by providing them with better training and the latest technology to reduce the broiler mortality rate. The companies should improve the development and introduction of high-quality chick breeds, thereby preventing extreme adverse events from the source. Second, since the intensity of the ratchet effect on historical peak mortality does not depend on the farmers' age or farming experience, but on the farming scale. Notably, due to habit formation, engaging traditional farmers in large-scale production whose main goal is to avoid risks, it is nearly impossible to reduce veterinary antibiotic use. The contract farming mode might be introduced to organize scattered small farmers' improved market entry and enhanced the surveillance of antibiotic use. Finally, breaking the link between antibiotic use and historical peak mortality is a key issue that the government and companies should focus on by revisiting the flaws and loopholes in the current policies. Especially, in low and middle-income countries where farmers are more sensitive to production risks and possess relatively low knowledge about disease management. After an extreme mortality event occurs, more technical guidance and support should be provided to farmers to improve their rational future disease risk management. Moreover, government could intervene to ensure producer risk transfer through the wider coverage of agricultural insurance in the poultry industry.

This study robustly answers the posited questions, yet it has some limitations, which offer avenues for future research. First, due to data constraints, it overlooks the differences between subtherapeutic and therapeutic antibiotic use. Second, the sampled company only records each farmer's total cost of antibiotics; albeit, most farmers cannot recall the dosage and stated that more than half of antibiotics were used for disease prevention. Thus, future research can accurately examines the cost of antibiotics used and incurred economic losses in mortality when no antibiotics were applied using control designs. Further, the sample data come from the same province in China and contract with the same breeding company; thus it might lack generalizability. Future research should include more farmers in other regions and contracting with different companies to verify the results represented herein. Further, although antibiotic use is discussed in this study, the conclusions can be extended to pesticide use both are damage control inputs. Subsequent research can directly investigate farmers' ratchet effect on pesticide use.

## Data availability statement

The raw data supporting the conclusions of this article will be made available by the authors, without undue reservation.

## Author contributions

LL: conceptualization, methodology, analysis, writing the original draft, and editing. RY: reviewing, supervision, and funding acquisition. All authors contributed to the article and approved the submitted version.

## Funding

This work was funded by National Natural Sciences Foundation of China (72073068 and 71573130), the Priority Academic Program Development of Jiangsu Higher Education Institutions, China (PAPD), and the China Center for Food Security Studies in Nanjing Agricultural University, China.

## Conflict of interest

The authors declare that the research was conducted in the absence of any commercial or financial relationships that could be construed as a potential conflict of interest.

## Publisher's note

All claims expressed in this article are solely those of the authors and do not necessarily represent those of their affiliated organizations, or those of the publisher, the editors and the reviewers. Any product that may be evaluated in this article, or claim that may be made by its manufacturer, is not guaranteed or endorsed by the publisher.

## Footnotes

^1^A threshold model should have been used to examine the threshold effect of historical peak mortality. However, the it requires balanced panel data, which result in the loss of a large number of samples because sampled farmers reared different batches of broilers. To be specific, as 18% of sample farmers reared no more than 4 batches of broilers, 25% reared 5–7 batches, 42% reared 8–10 batches, and the left 15% reared 11–14 batches, at least half of the sampled would be lost. Therefore, we choose to create a series of dummy variables to measure the threshold effect. To verify the robustness of the threshold effect, we also adopted a fixed-effect panel threshold model proposed by Hansen (42). To reduce sample attrition, farmers that reared 8 and more batches were kept and batches above 8 were dropped. We first test for one, two and three-threshold models through a bootstrap procedure. The test statistics suggest the presence of one significant threshold value, which is 0.143. With the estimated threshold values, a fixed-effect panel threshold model is estimated. Results show that when the historical peak mortality is lower than 0.143, the coefficient of historical peak mortality on antibiotic use is 0.135 and significant at the 10% level. On the other hand, if the historical peak mortality is higher than 0.143, the coefficient of historical peak mortality on antibiotic use increases to 0.307 and is significant at the 1% level. By contrast, the impact of historical peak mortality on antibiotic use is much greater when historical peak mortality is relatively high. In general, the panel threshold model yields consistent estimates with the analysis that uses a series of dummy variables. As the sample size is cut (at least halved) when using the panel threshold model, we stick with the estimation with a series of dummy variables. Due to space limitations, detailed results are not reported and can be obtained from the authors.
